# Supplementary material for: Climate-induced forest dieback drives compositional changes in insect communities that are more pronounced for rare species
Source: Commun Biol. 2022 Jan 18;5:57. doi: 10.1038/s42003-021-02968-4 (PMC8766456; doi:10.1038/s42003-021-02968-4)
Supplement: Supplementary file 2 — Supplementary Information [file 42003_2021_2968_MOESM2_ESM.pdf]

Climate-induced forest dieback drives compositional changes in insect communities that are more pronounced for rare species

Supplementary Information – Figures and Tables

Summary

Supplementary Figures *2*

Supplementary Figure 1 2

Supplementary Figure 2 3

Supplementary Figure 3 5

Supplementary Figure 4 6

Supplementary Figure 5 7

Supplementary Figure 6 9

Supplementary Tables *11*

Supplementary Table 1 11

Supplementary Table 2 13

Supplementary Figures

Supplementary Figure 1: **Observed and estimated insect MOTUs richness.**

MOTUs richness observed and estimated, based on Chao2 and Jack1 (first order jackknife) analyses for the 56 plots. While the recovered insect diversity of the present study is of 2972 MOTUs, both estimation methods give around 4000 MOTUs trappable using Malaise trap on the four-months sampling period considered.

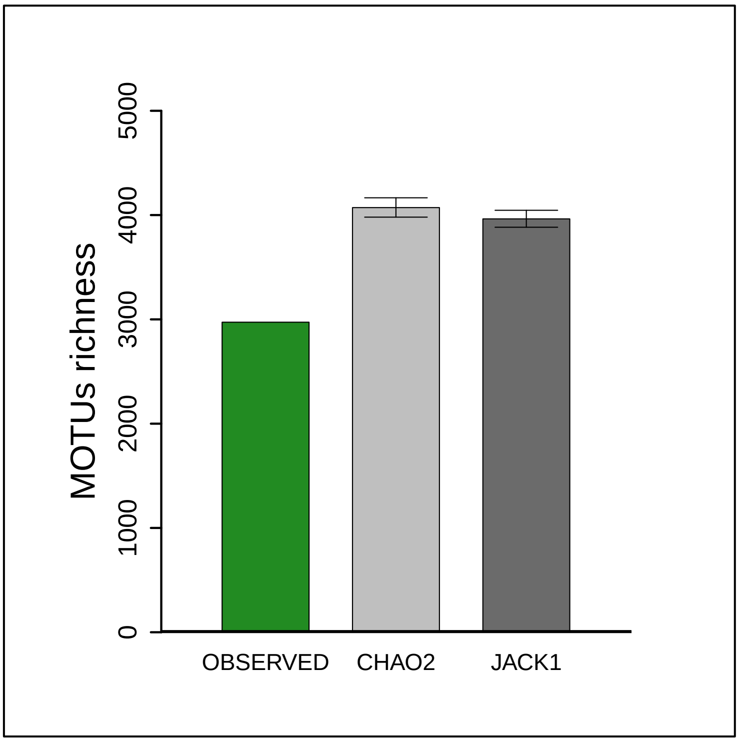

Supplementary Figure 2: **Relative MOTUs prevalence per dieback category and sampled district.**

Heatmaps of MOTU prevalence (proportion of samples in which a MOTU is present) by (**a**) geographical district and (**b**) dieback category. The incidence dataset was bootstrapped 100 times with plot randomization for a total of 5600 virtual samples. Bootstrapped plots were concatenated per (**a**) respective sampled districts or (**b**) dieback categories. For (**a**), prevalence per MOTU is shown per district. Thus, bright yellow (100%) in one district indicates a complete presence of the MOTU in the respective district or in the total dataset. For (**b**), prevalence per MOTU is shown in percentage scaled per dieback category to correspond to 1/3 of the potential 100% maximum presence across all categories. Thus, bright red (33%) in one category means the MOTU is present in 100% of the sampled plots of this respective category. MOTUs can be present at 33% in each category for a total of 100% presence in the complete dataset. Here, we observe that common MOTUs across (**a**) sampled districts or (**b**) dieback categories are the most prevalent and that specific community compositions are mainly shaped by rarely occurring MOTUs.

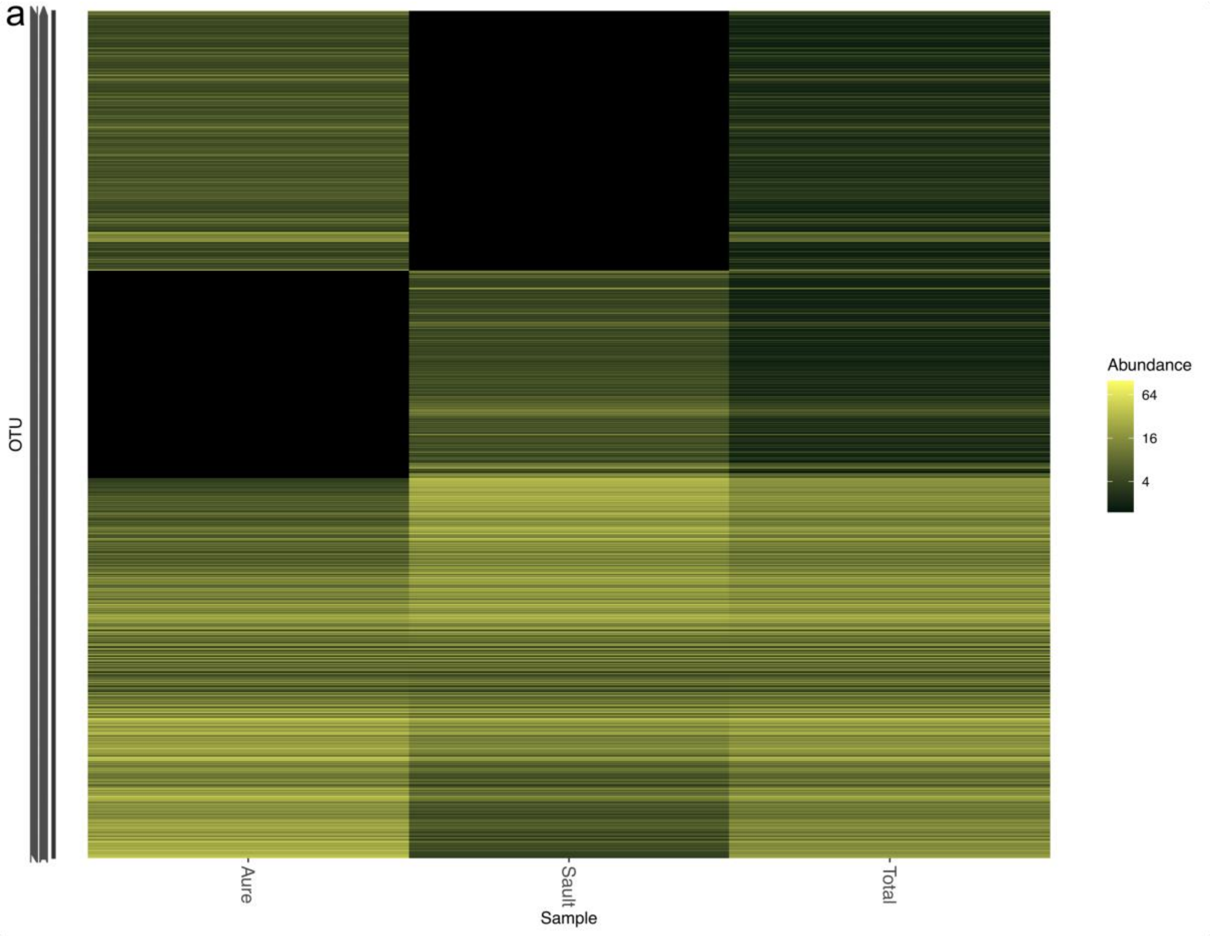

b

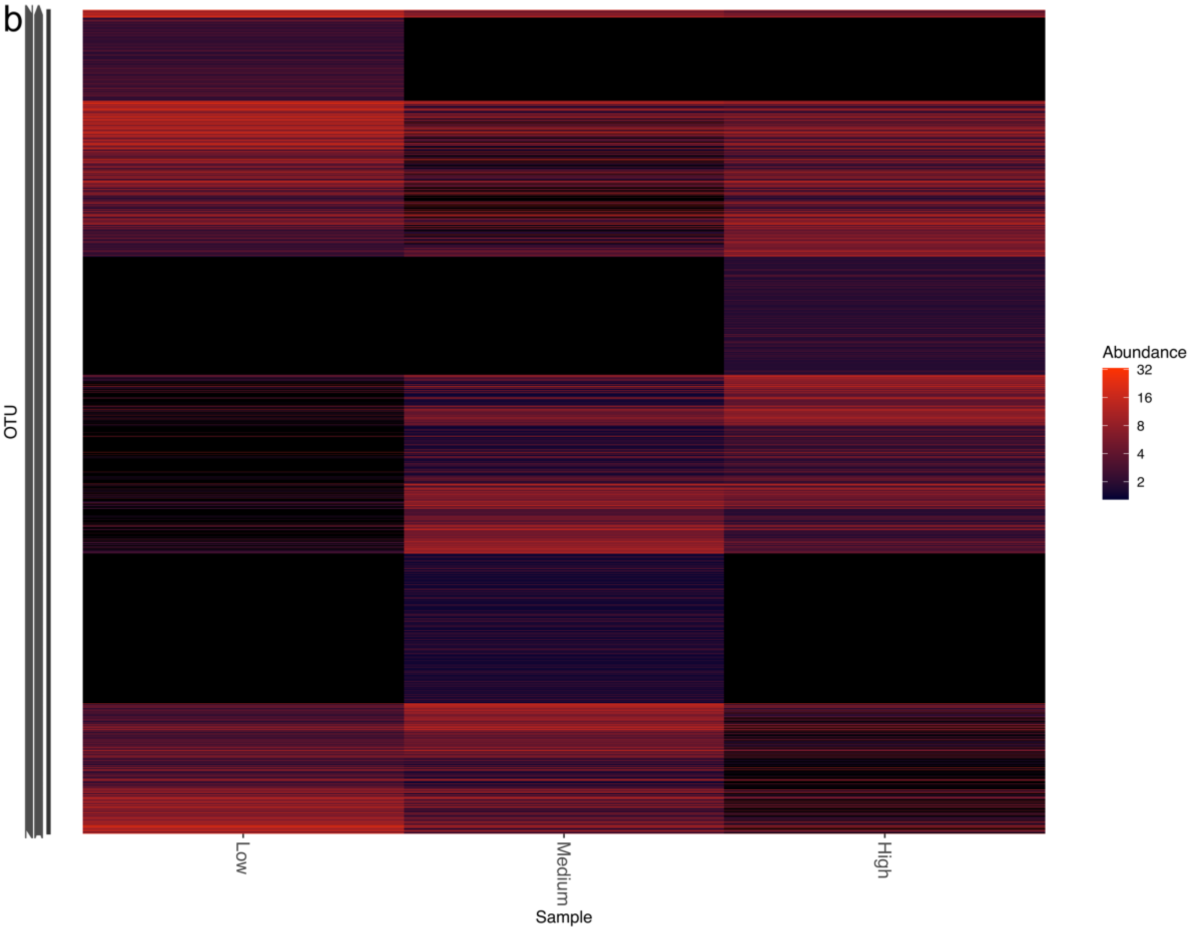

Supplementary Figure 3: **DNA-based taxonomic identification coverage for the five most represented insect orders.**

Dark green bars show the total number of MOTUs recovered for each of the five most represented insect orders (Diptera, Hymenoptera, Lepidoptera, Coleoptera and Hemiptera). Grey-blue, blue and and light-blue bars represent the number of MOTUs for each order that has been associated with no ambiguity to family, genus and species level, respectively. Taxonomic assignment was performed using BOLD System DNA reference libraries from April 2019.

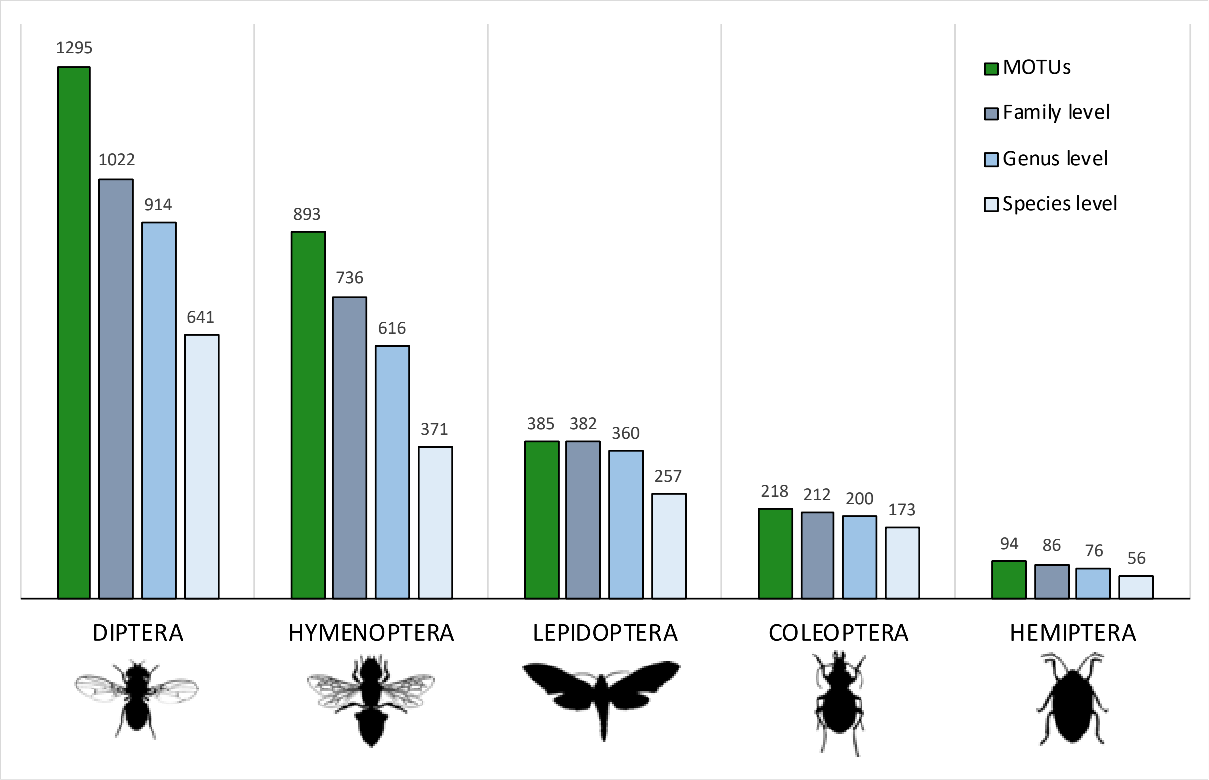

Supplementary Figure 4: **Zeta-diversity decline and model fitting on the insect fauna for all combinations and assemblages.**

Zeta-diversity analyses per Zeta order (*i.e.* ζ_i) here referring to plots (from $\zeta2$ to $\zeta56$) for two computing schemes. Representations consider non-geographically structured scheme that computes all combinations and assemblages (ALL) with parameter sample set to 5000 and Monte-Carlo (mc) sampling. (**a, b**) Zeta-diversity decline and 0 to 1 scaled ratio of Zeta diversity decline, representing species shared and species retention rate (*i.e.* the retention probability of common species in the community) across ζ_i, respectively. (**c, d**) Zeta-diversity model fitting to exponential and power-law regressions, respectively.

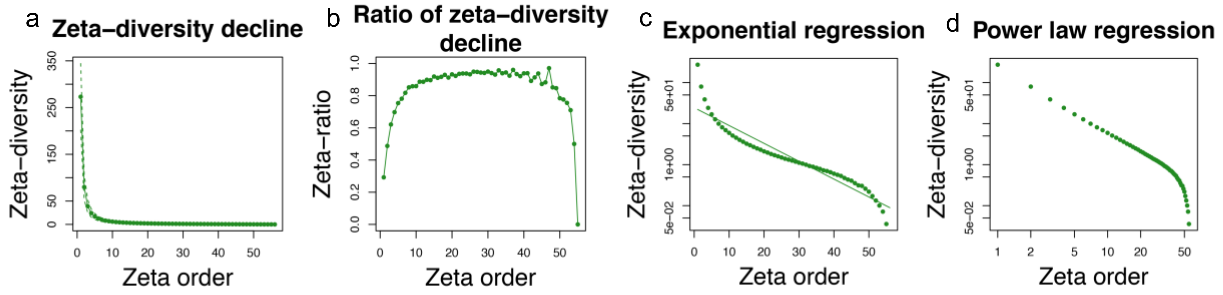

Supplementary Figure 5: **Effect of dieback on community composition for insect orders and functions.**

Representation of the species retention rate (*i.e.* zeta ratio) per plot (*i.e.* Zeta order) following all plot combinations scheme (ALL) with parameter sample set to 5000 and Monte-Carlo (mc) sampling for low, medium and high dieback levels, respectively (**a, b, c**) for the five main insect Orders (Coleoptera, Diptera, Hemiptera, Hymenoptera and Lepidoptera) and (**d, e, f**) for the four main ecological functions recovered from taxonomic assignment (floricolous / non floricolous and parasitoid / non-parasitoid species). Green line with plain dots represents mean species retention rate of the total dataset in each respective dieback category. Increasing curves express that common MOTUs are more likely to be retained in additional samples than rare ones (with presence of common species over all plots if zeta ratio = 1) and decreasing curves indicates species turnover.

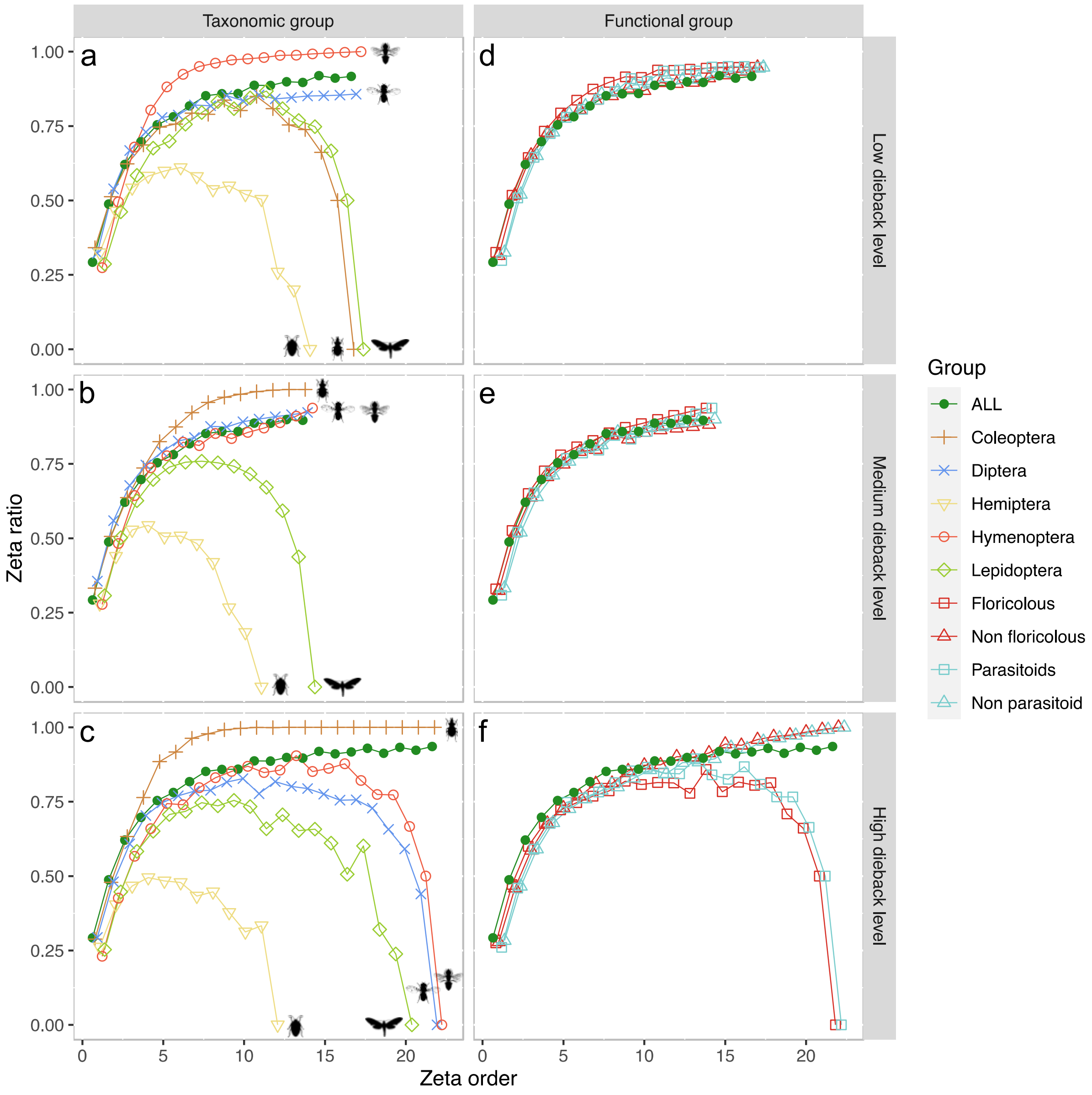

Taxonomic group
Functional group
Low dieback level
Medium dieback level
High dieback level
Zeta ratio
Zeta order
a
b
c
d
e
f
1.00
0.75
0.50
0.25
0.00
0
5
10
15
20
Group
ALL
Coleoptera
Diptera
Hemiptera
Hymenoptera
Lepidoptera
Floricolous
Non floricolous
Parasitoids
Non parasitoid

Supplementary Figure 6: **Effect of stand type on community composition for insect orders and functions.**

Representation of the species retention rate (*i.e.* zeta ratio) per plot (*i.e.* Zeta order) following all plot combinations scheme (ALL) with parameter sample set to 5000 and Monte-Carlo (mc) sampling for healthy, disturbed and salvaged stands, respectively (**a, b, c**) for the five main insect Orders (Coleoptera, Diptera, Hemiptera, Hymenoptera and Lepidoptera) and (**d, e, f**) for the four main ecological functions recovered from taxonomic assignment (floricolous / non floricolous and parasitoid / non-parasitoid species). Green line with plain dots represents mean species retention rate of the total dataset in each respective dieback category. Increasing curves express that common MOTUs are more likely to be retained in additional samples than rare ones (with presence of common species over all plots if zeta ratio = 1) and decreasing curves indicates species turnover.

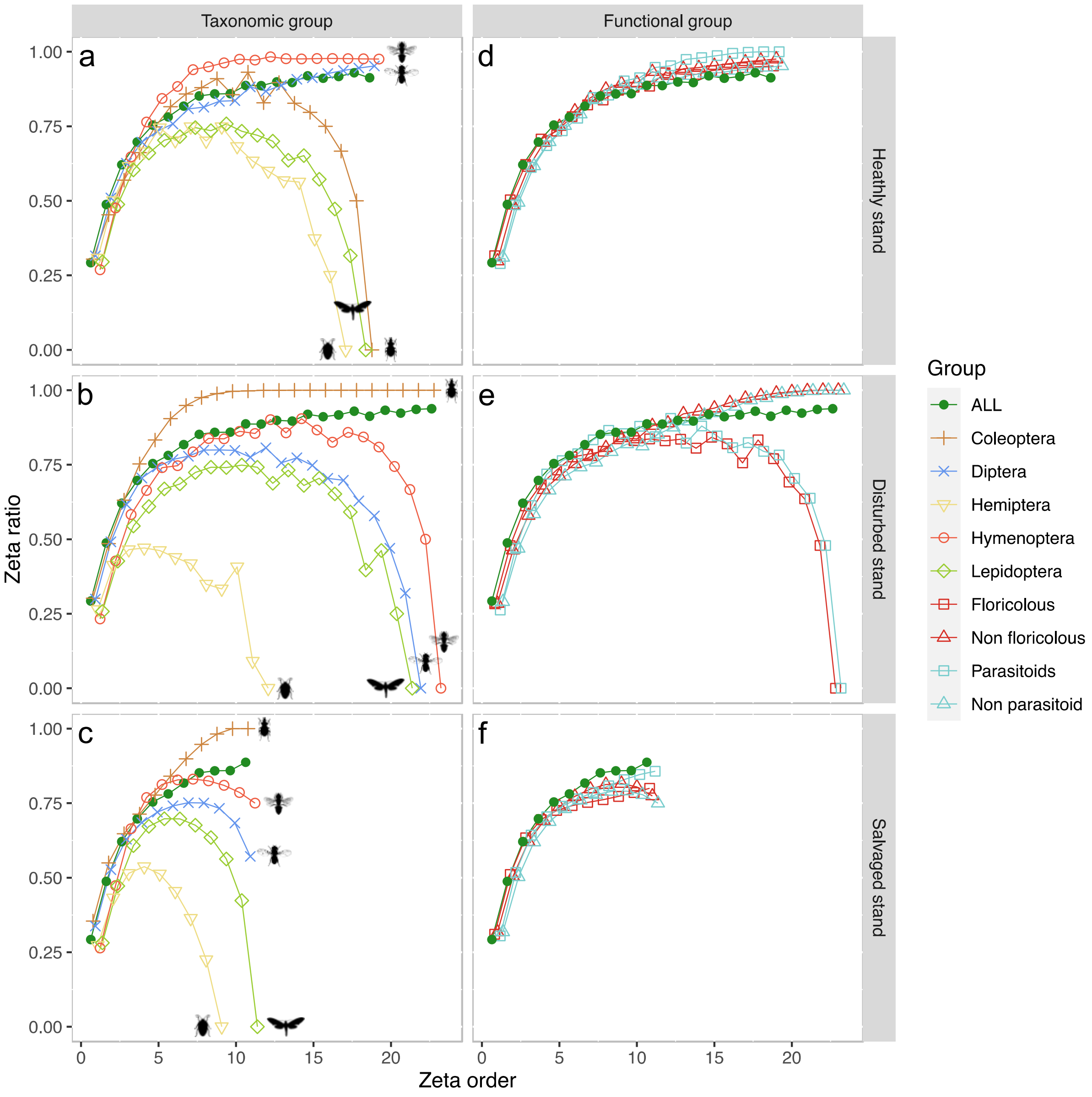
Taxonomic group
Functional group
a
b
c
d
e
f
Heathly stand
Disturbed stand
Salvaged stand
Zeta ratio
Zeta order
1.00
0.75
0.50
0.25
0.00
0
5
10
15
20
Group
ALL
Coleoptera
Diptera
Hemiptera
Hymenoptera
Lepidoptera
Floricolous
Non floricolous
Parasitoids
Non parasitoid

Supplementary Tables

Supplementary Table 1: **Impact of forest dieback and salvage logging on insect communities.**

Generalized linear models from mvabund object for community composition changes of different study groups (*i.e.* total insects, five most represented insect orders, and four functional group) compared across respective environmental conditions of diebacks (*i.e.* low, medium and high forest dieback levels) and stand types (*i.e.* healthy, disturbed and salvaged logged. Functional groups (*i.e.* parasitoid/non-parasitoid, floricolous/non-floricolous insects) were assigned using each MOTU's taxonomic family. Reported values are the two extremes given from 10 consecutive analyses. Post-Hoc Holm correction was applied on the complete 10 trials range of each group. Significance is given by "*****" with relative values in bold, while "*N.S.*" stands for non-significant. We highlight that all studied groups but Coleoptera had community composition changes driven by forest dieback. Significant compositional changes also arose in each case between study districts.

Studied group	Variable effect	Effect' range of p-values (95% confidence)	Post-Hoc Holm correction	Significance
**Total insects**				
	Forest dieback	0.007 to 0.001	**0.024 to 0.018**	***** to ******
	Stand type	0.062 to 0.049	0.49	*N.S.*
	District	0.001	**0.01**	******
**Coleoptera**				
	Forest dieback	0.043 to 0.024	0.27	*N.S.*
	Stand type	0.210 to 0.163	1	*N.S.*
	District	0.001	**0.01**	******
**Diptera**				
	Forest dieback	0.005 to 0.002	**0.028 to to 0.02**	*****
	Stand type	0.149 to 0.109	1	*N.S.*
	District	0.001	**0.01**	******
**Hemiptera**				
	Forest dieback	0.008 to 0.002	**0.036 to 0.02**	*****
	Stand type	0.205 to 0.176	1	*N.S.*
	District	0.001	**0.01**	******
**Hymenoptera**				
	Forest dieback	0.007 to 0.001	**0.03 to 0.01**	***** to ******
	Stand type	0.042 to 0.027	0.28	*N.S.*
	District	0.001	**0.01**	******
**Lepidoptera**				
	Forest dieback	0.011 to 0.003	0.064 to **0.03**	*N.S.* to *****
	Stand type	0.025 to 0.009	0.144 to 0.09	*N.S.*
	District	0.001	**0.01**	******
**Floricolous**				
	Forest dieback	0.007 to 0.003	**0.03**	*****
	Stand type	0.056 to 0.038	0.38	*N.S.*
	District	0.001	**0.01**	******
**Non floricolous**				
	Forest dieback	0.004 to 0.001	**0.016** to **0.01**	******
	Stand type	0.08 to 0.06	0.6	*N.S.*
	District	0.001	**0.01**	******
**Parasitoid**				
	Forest dieback	0.008 to 0.001	**0.024** to **0.01**	***** to ******
	Stand type	0.033 to 0.016	0.26 to 0.16	*N.S.*
	District	0.001	**0.01**	******
**Non parasitoid**				
	Forest dieback	0.006 to 0.002	**0.024** to **0.02**	*****
	Stand type	0.091 to 0.063	0.63	*N.S.*
	District	0.001	**0.01**	******

Supplementary Table 2: **Species associated with a specific dieback level.**

Table of IndVal analysis to determine MOTUs significantly associated to each dieback level (*i.e.* low, medium and high). IndVal analyses were performed 10 times and only MOTUs with 10 significant associations for each dieback level were retained. Post-hoc Holm correction was then applied on the 10 p-values range reported for each MOTU. Significance is given by "*****" with relative values in bold, while "*N.S.*" stands for non-significant.

Taxonomy	MOTUs	Uncorrected										Holm correction										Significance	Dieback level
		RUN 1	RUN 2	RUN 3	RUN 4	RUN 5	RUN 6	RUN 7	RUN 8	RUN 9	RUN 10	RUN 1	RUN 2	RUN 3	RUN 4	RUN 5	RUN 6	RUN 7	RUN 8	RUN 9	RUN 10		
Entiminae sp.	MOTU1110	0.025	0.016	0.019	0.024	0.018	0.019	0.027	0.021	0.025	0.026	0.162	0.16	0.162	0.162	0.162	0.162	0.162	0.162	0.162	0.162	*N.S.*	LOW
Peyerimhoffina gracilis	MOTU1115	0.005	0.005	0.003	0.005	0.003	0.01	0.002	0.007	0.003	0.002	**0.025**	**0.025**	**0.024**	**0.025**	**0.024**	**0.025**	**0.02**	**0.025**	**0.024**	**0.02**	* to **	
Cecidomyiidae sp.	MOTU1127	0.002	0.001	0.001	0.002	0.003	0.004	0.002	0.005	0.005	0.003	**0.016**	**0.01**	**0.01**	**0.016**	**0.016**	**0.016**	**0.016**	**0.016**	**0.016**	**0.016**	**	
Diptera sp.	MOTU1155	0.005	0.004	0.005	0.005	0.008	0.004	0.005	0.009	0.009	0.01	**0.04**	**0.04**	**0.04**	**0.04**	**0.04**	**0.04**	**0.04**	**0.04**	**0.04**	**0.04**	*	
Diptera sp.	MOTU1168	0.012	0.012	0.014	0.016	0.017	0.012	0.015	0.009	0.008	0.015	0.096	0.096	0.096	0.096	0.096	0.096	0.096	0.081	0.08	0.096	*N.S.*	
Enclisis ornaticeps	MOTU1169	0.022	0.025	0.011	0.021	0.019	0.019	0.012	0.018	0.028	0.025	0.144	0.144	0.11	0.144	0.144	0.144	0.11	0.144	0.144	0.144	*N.S.*	
Hyperlasion wasmanni	MOTU120	0.003	0.002	0.002	0.005	0.005	0.003	0.002	0.002	0.001	0.001	**0.016**	**0.016**	**0.016**	**0.016**	**0.016**	**0.016**	**0.016**	**0.016**	**0.01**	**0.01**	**	
Diptera sp.	MOTU1255	0.016	0.021	0.015	0.019	0.021	0.019	0.017	0.018	0.014	0.017	0.14	0.14	0.14	0.14	0.14	0.14	0.14	0.14	0.14	0.14	*N.S.*	
Calobatinae sp.	MOTU1257	0.016	0.016	0.018	0.02	0.017	0.012	0.023	0.014	0.014	0.014	0.126	0.126	0.126	0.126	0.126	0.12	0.126	0.126	0.126	0.126	*N.S.*	
Rhinophora lepida	MOTU134	0.02	0.015	0.01	0.016	0.015	0.013	0.019	0.009	0.017	0.025	0.105	0.105	0.09	0.105	0.105	0.104	0.105	0.09	0.105	0.105	*N.S.*	
Temnothorax affinis	MOTU1437	0.015	0.016	0.017	0.023	0.025	0.012	0.014	0.011	0.018	0.015	0.112	0.112	0.112	0.112	0.112	0.11	0.112	0.11	0.112	0.112	*N.S.*	
Diptera sp.	MOTU1440	0.01	0.006	0.008	0.004	0.013	0.006	0.006	0.008	0.005	0.007	**0.048**	**0.048**	**0.048**	**0.04**	**0.048**	**0.048**	**0.048**	**0.048**	**0.045**	**0.048**	*	
Aulonothroscus brevicollis	MOTU1501	0.008	0.004	0.003	0.005	0.006	0.01	0.007	0.009	0.009	0.009	**0.042**	**0.036**	**0.03**	**0.04**	**0.042**	**0.042**	**0.042**	**0.042**	**0.042**	**0.042**	*	
Sphegina clunipes	MOTU1561	0.019	0.01	0.011	0.009	0.014	0.013	0.015	0.013	0.01	0.018	0.09	0.09	0.09	0.09	0.09	0.09	0.09	0.09	0.09	0.09	*N.S.*	
Diptera sp.	MOTU170	0.043	0.047	0.044	0.042	0.043	0.046	0.038	0.034	0.031	0.037	0.31	0.31	0.31	0.31	0.31	0.31	0.31	0.31	0.31	0.31	*N.S.*	
Peripsocus cf. didymus	MOTU1988	0.02	0.013	0.019	0.019	0.018	0.019	0.015	0.013	0.014	0.016	0.13	0.13	0.13	0.13	0.13	0.13	0.13	0.13	0.13	0.13	*N.S.*	
Philotarsus picicornis	MOTU2018	0.007	0.006	0.005	0.006	0.006	0.005	0.008	0.008	0.005	0.007	**0.05**	**0.05**	**0.05**	**0.05**	**0.05**	**0.05**	**0.05**	**0.05**	**0.05**	**0.05**	*	
Chironomus luridus	MOTU2093	0.02	0.016	0.018	0.018	0.015	0.014	0.02	0.019	0.016	0.021	0.14	0.14	0.14	0.14	0.14	0.14	0.14	0.14	0.14	0.14	*N.S.*	
Diptera sp.	MOTU2365	0.012	0.023	0.015	0.018	0.015	0.025	0.019	0.019	0.017	0.012	0.12	0.12	0.12	0.12	0.12	0.12	0.12	0.12	0.12	0.12	*N.S.*	
Diptera sp.	MOTU2616	0.013	0.017	0.019	0.012	0.018	0.026	0.016	0.021	0.016	0.018	0.12	0.128	0.128	0.12	0.128	0.128	0.128	0.128	0.128	0.128	*N.S.*	
Megaselia verna	MOTU2713	0.011	0.009	0.013	0.016	0.013	0.009	0.008	0.012	0.013	0.011	0.081	0.081	0.081	0.081	0.081	0.081	0.08	0.081	0.081	0.081	*N.S.*	
Apodesmia incisula	MOTU2966	0.013	0.01	0.014	0.014	0.013	0.016	0.011	0.01	0.012	0.014	0.1	0.1	0.1	0.1	0.1	0.1	0.1	0.1	0.1	0.1	*N.S.*	
Hymenoptera sp.	MOTU308	0.005	0.004	0.003	0.002	0.006	0.005	0.003	0.004	0.007	0.007	**0.028**	**0.028**	**0.027**	**0.02**	**0.028**	**0.028**	**0.027**	**0.028**	**0.028**	**0.028**	*	
Herminia tarsipennalis	MOTU333	0.012	0.023	0.017	0.019	0.015	0.026	0.017	0.016	0.013	0.019	0.12	0.12	0.12	0.12	0.12	0.12	0.12	0.12	0.12	0.12	*N.S.*	
Coenosia sallae	MOTU348	0.003	0.002	0.004	0.007	0.007	0.005	0.005	0.005	0.004	0.006	**0.027**	**0.02**	**0.032**	**0.032**	**0.032**	**0.032**	**0.032**	**0.032**	**0.032**	**0.032**	* to **	
Diptera sp.	MOTU371	0.03	0.013	0.017	0.024	0.021	0.021	0.015	0.026	0.023	0.028	0.147	0.13	0.136	0.147	0.147	0.147	0.135	0.147	0.147	0.147	*N.S.*	
Eilema sp.	MOTU39	0.001	0.001	0.001	0.001	0.001	0.001	0.001	0.001	0.001	0.001	**0.01**	**0.01**	**0.01**	**0.01**	**0.01**	**0.01**	**0.01**	**0.01**	**0.01**	**0.01**	**	
Peromyia sp.	MOTU472	0.003	0.005	0.004	0.004	0.008	0.006	0.005	0.004	0.005	0.005	**0.03**	**0.036**	**0.036**	**0.036**	**0.036**	**0.036**	**0.036**	**0.036**	**0.036**	**0.036**	*	
Hymenoptera sp.	MOTU474	0.003	0.003	0.003	0.003	0.002	0.002	0.003	0.002	0.003	0.002	**0.02**	**0.02**	**0.02**	**0.02**	**0.02**	**0.02**	**0.02**	**0.02**	**0.02**	**0.02**	**	
Platylabus sp.	MOTU647	0.017	0.01	0.012	0.02	0.009	0.008	0.02	0.011	0.011	0.011	0.081	0.081	0.081	0.081	0.081	0.08	0.081	0.081	0.081	0.081	*N.S.*	
Stenomacrus celer	MOTU710	0.005	0.005	0.004	0.009	0.005	0.004	0.003	0.003	0.006	0.004	**0.032**	**0.032**	**0.032**	**0.032**	**0.032**	**0.032**	**0.03**	**0.03**	**0.032**	**0.032**	*	
Phaonia sp.	MOTU754	0.018	0.009	0.022	0.023	0.032	0.019	0.028	0.025	0.025	0.031	0.162	0.09	0.162	0.162	0.162	0.162	0.162	0.162	0.162	0.162	*N.S.*	
Diptera sp.	MOTU80	0.007	0.004	0.011	0.007	0.013	0.006	0.008	0.008	0.009	0.012	0.056	**0.04**	0.056	0.056	0.056	0.054	0.056	0.056	0.056	0.056	*N.S. to* *	
Cicadellidae sp.	MOTU868	0.001	0.001	0.001	0.001	0.001	0.001	0.001	0.001	0.001	0.001	0.01	0.01	0.01	0.01	0.01	0.01	0.01	0.01	0.01	0.01	**	
Lasius platythorax	MOTU870	0.014	0.015	0.021	0.029	0.019	0.011	0.016	0.014	0.024	0.017	0.126	0.126	0.126	0.126	0.126	0.11	0.126	0.126	0.126	0.126	*N.S.*	
Glyphipterix forsterella	MOTU922	0.005	0.017	0.02	0.019	0.019	0.011	0.01	0.02	0.016	0.012	**0.05**	0.096	0.096	0.096	0.096	0.09	0.09	0.096	0.096	0.09	*N.S. to* *	
Lethades facialis	MOTU1047	0.015	0.016	0.015	0.017	0.014	0.02	0.019	0.013	0.015	0.017	0.13	0.13	0.13	0.13	0.13	0.13	0.13	0.13	0.13	0.13	*N.S.*	MEDIUM
Compsilura concinnata	MOTU1108	0.011	0.007	0.006	0.003	0.002	0.008	0.003	0.006	0.004	0.005	**0.03**	**0.03**	**0.03**	**0.027**	**0.02**	**0.03**	**0.027**	**0.03**	**0.028**	**0.03**	*	
Argyresthia conjugella	MOTU1193	0.01	0.017	0.019	0.025	0.011	0.013	0.013	0.011	0.015	0.008	0.09	0.09	0.09	0.09	0.09	0.09	0.09	0.09	0.09	0.08	*N.S.*	
Diptera sp.	MOTU1300	0.033	0.035	0.037	0.025	0.034	0.028	0.037	0.037	0.046	0.029	0.252	0.252	0.252	0.25	0.252	0.252	0.252	0.252	0.252	0.252	*N.S.*	
Lonchaea postica	MOTU1320	0.02	0.031	0.027	0.024	0.018	0.028	0.023	0.025	0.032	0.027	0.18	0.184	0.184	0.184	0.18	0.184	0.184	0.184	0.184	0.184	*N.S.*	
Stromboceros delicatulus	MOTU1366	0.015	0.015	0.012	0.01	0.012	0.009	0.016	0.01	0.008	0.007	0.072	0.072	0.072	0.072	0.072	0.072	0.072	0.072	0.072	0.07	*N.S.*	
Passaloecus insignis	MOTU1504	0.044	0.041	0.035	0.043	0.046	0.039	0.045	0.039	0.034	0.038	0.34	0.34	0.34	0.34	0.34	0.34	0.34	0.34	0.34	0.34	*N.S.*	
Tipula scripta	MOTU1535	0.014	0.013	0.017	0.016	0.015	0.022	0.027	0.015	0.01	0.011	0.104	0.104	0.104	0.104	0.104	0.104	0.104	0.104	0.1	0.1	*N.S.*	
Perilissus rufoniger	MOTU1671	0.021	0.012	0.018	0.015	0.02	0.022	0.022	0.017	0.021	0.017	0.136	0.12	0.136	0.135	0.136	0.136	0.136	0.136	0.136	0.136	*N.S.*	
Hylaeus confusus	MOTU2070	0.019	0.014	0.021	0.021	0.019	0.017	0.018	0.02	0.019	0.029	0.153	0.14	0.153	0.153	0.153	0.153	0.153	0.153	0.153	0.153	*N.S.*	
Diplazon sp.	MOTU2274	0.012	0.027	0.016	0.015	0.012	0.023	0.017	0.018	0.019	0.019	0.12	0.12	0.12	0.12	0.12	0.12	0.12	0.12	0.12	0.12	*N.S.*	
Trichosia edwardsi	MOTU2607	0.007	0.006	0.006	0.002	0.007	0.008	0.002	0.006	0.001	0.008	**0.042**	**0.042**	**0.042**	**0.018**	**0.042**	**0.042**	**0.018**	**0.042**	**0.01**	**0.042**	* to **	
Lyciella rorida	MOTU261	0.038	0.024	0.029	0.026	0.039	0.029	0.027	0.027	0.03	0.034	0.24	0.24	0.24	0.24	0.24	0.24	0.24	0.24	0.24	0.24	*N.S.*	
Eustalomyia festiva	MOTU278	0.025	0.016	0.01	0.021	0.015	0.02	0.016	0.023	0.021	0.025	0.135	0.135	0.1	0.135	0.135	0.135	0.135	0.135	0.135	0.135	*N.S.*	
Scathophaga sp.	MOTU292	0.043	0.029	0.034	0.039	0.027	0.026	0.024	0.032	0.033	0.027	0.24	0.24	0.24	0.24	0.24	0.24	0.24	0.24	0.24	0.24	*N.S.*	
Macquartia dispar	MOTU318	0.017	0.016	0.022	0.024	0.025	0.024	0.028	0.019	0.016	0.022	0.16	0.16	0.16	0.16	0.16	0.16	0.16	0.16	0.16	0.16	*N.S.*	
Leptosciarella scutellata	MOTU322	0.019	0.026	0.021	0.024	0.027	0.018	0.017	0.025	0.022	0.017	0.17	0.17	0.17	0.17	0.17	0.17	0.17	0.17	0.17	0.17	*N.S.*	
Melinda sp.	MOTU349	0.021	0.037	0.026	0.038	0.047	0.037	0.036	0.027	0.033	0.042	0.21	0.234	0.234	0.234	0.234	0.234	0.234	0.234	0.234	0.234	*N.S.*	
Volucella sp.	MOTU420	0.005	0.007	0.006	0.005	0.002	0.007	0.008	0.003	0.006	0.006	**0.04**	**0.04**	**0.04**	**0.04**	**0.02**	**0.04**	**0.04**	**0.027**	**0.04**	**0.04**	*	
Syrphinae sp.	MOTU44	0.014	0.016	0.016	0.013	0.016	0.009	0.017	0.016	0.019	0.018	0.117	0.117	0.117	0.117	0.117	0.09	0.117	0.117	0.117	0.117	*N.S.*	
Tenthredo sp.	MOTU442	0.013	0.01	0.009	0.015	0.008	0.008	0.012	0.012	0.01	0.007	0.072	0.072	0.072	0.072	0.072	0.072	0.072	0.072	0.072	0.07	*N.S.*	
Phaonia angelicae	MOTU47	0.002	0.003	0.002	0.002	0.002	0.001	0.003	0.001	0.001	0.003	0.014	0.014	0.014	0.014	0.014	0.01	0.014	0.01	0.01	0.014	**	
Phaonia rufiventris	MOTU495	0.019	0.017	0.022	0.018	0.014	0.018	0.013	0.014	0.013	0.01	0.117	0.117	0.117	0.117	0.117	0.117	0.117	0.117	0.117	0.1	*N.S.*	
Hymenoptera sp.	MOTU660	0.004	0.004	0.004	0.002	0.006	0.005	0.006	0.004	0.004	0.009	**0.036**	**0.036**	**0.036**	**0.02**	**0.036**	**0.036**	**0.036**	**0.036**	**0.036**	**0.036**	* to **	
Pieris sp.	MOTU688	0.012	0.02	0.014	0.014	0.019	0.016	0.018	0.015	0.013	0.024	0.12	0.12	0.12	0.12	0.12	0.12	0.12	0.12	0.12	0.12	*N.S.*	
Eristalis pertinax	MOTU725	0.015	0.016	0.023	0.023	0.028	0.014	0.028	0.021	0.016	0.022	0.14	0.14	0.14	0.14	0.14	0.14	0.14	0.14	0.14	0.14	*N.S.*	
Suillia ustulata	MOTU729	0.027	0.031	0.03	0.035	0.029	0.038	0.03	0.021	0.03	0.034	0.243	0.243	0.243	0.243	0.243	0.243	0.243	0.21	0.243	0.243	*N.S.*	
Bradysia placida	MOTU826	0.029	0.035	0.044	0.048	0.047	0.045	0.043	0.049	0.049	0.047	0.29	0.315	0.344	0.344	0.344	0.344	0.344	0.344	0.344	0.344	*N.S.*	
Muscina pascuorum	MOTU937	0.014	0.017	0.013	0.017	0.013	0.017	0.02	0.016	0.02	0.013	0.13	0.13	0.13	0.13	0.13	0.13	0.13	0.13	0.13	0.13	*N.S.*	
Dusona sp.	MOTU997	0.023	0.024	0.016	0.019	0.013	0.022	0.019	0.011	0.016	0.025	0.128	0.128	0.128	0.128	0.117	0.128	0.128	0.11	0.128	0.128	*N.S.*	
Polistes sp.	MOTU1093	0.013	0.011	0.014	0.013	0.011	0.013	0.013	0.011	0.007	0.016	0.099	0.099	0.099	0.099	0.099	0.099	0.099	0.099	0.07	0.099	*N.S.*	HIGH
Dichrogaster sp.	MOTU1104	0.032	0.03	0.033	0.025	0.033	0.034	0.033	0.036	0.036	0.038	0.27	0.27	0.27	0.25	0.27	0.27	0.27	0.27	0.27	0.27	*N.S.*	
Raphidioptera	MOTU1229	0.038	0.033	0.038	0.038	0.041	0.05	0.037	0.02	0.034	0.035	0.297	0.297	0.297	0.297	0.297	0.297	0.297	0.2	0.297	0.297	*N.S.*	
Diptera sp.	MOTU1471	0.036	0.032	0.037	0.036	0.036	0.024	0.033	0.036	0.032	0.03	0.27	0.27	0.27	0.27	0.27	0.24	0.27	0.27	0.27	0.27	*N.S.*	
Spelobia quinata	MOTU1954	0.006	0.01	0.014	0.01	0.018	0.013	0.012	0.006	0.011	0.012	0.06	0.08	0.08	0.08	0.08	0.08	0.08	0.06	0.08	0.08	*N.S.*	
Lymnophyes sp.	MOTU2297	0.038	0.026	0.05	0.033	0.041	0.041	0.027	0.04	0.032	0.032	0.26	0.26	0.26	0.26	0.26	0.26	0.26	0.26	0.26	0.26	*N.S.*	
Scaeva sp.	MOTU26	0.038	0.039	0.044	0.039	0.041	0.037	0.037	0.035	0.034	0.037	0.34	0.34	0.34	0.34	0.34	0.34	0.34	0.34	0.34	0.34	*N.S.*	
Aphantopus hyperantus	MOTU876	0.039	0.039	0.039	0.038	0.039	0.037	0.044	0.037	0.039	0.035	0.35	0.35	0.35	0.35	0.35	0.35	0.35	0.35	0.35	0.35	*N.S.*	
