# Supplementary material for: Climate-induced forest dieback drives compositional changes in insect communities that are more pronounced for rare species
Source: Commun Biol. 2022 Jan 18;5:57. doi: 10.1038/s42003-021-02968-4 (PMC8766456; doi:10.1038/s42003-021-02968-4)
Supplement: Supplementary file 3 — Description of Additional Supplementary Files [file 42003_2021_2968_MOESM3_ESM.pdf]

Description of Additional Supplementary Files

File name: Supplementary Data 1.
Description: List of the 2972 MOTUs recovered from metabarcoding analyses and considered in the present study.

Each MOTUs recovered with DNA consensus sequences and taxonomic information for each MOTU and their presence or absence in each sampled plot. Taxonomy was

recovered using a blast search in BOLD with a 97% threshold; NA stands for conflicting matches or in the absence of a match. For each sampled plot, presence (1) / absence (0) value from the total grouping of the four 1- month samplings is reported. Red columns are samples lost or that did not yield result after demultiplexing.

File name: Supplementary Data 2.
Description: List of the 258 insect families recovered from metabarcoding analyses and their assigned ecological functions.

Each family was assigned to four main functional groups: floricolous / non floricolous based on adult trophic guilds and parasitoid / non parasitoid based on larval trophic guilds. "Yes/no" case refers to two species from a family were recovered and where functions could be distinguished. "NA" cases stands for not available or unknown results.

File name: Supplementary Data 3.
Description: Plot list.

File name: Supplementary Data 4.
Description: Primer list.
