# Supplementary material for: Climate-induced forest dieback drives compositional changes in insect communities that are more pronounced for rare species
Source: Commun Biol. 2022 Jan 18;5:57. doi: 10.1038/s42003-021-02968-4 (PMC8766456; doi:10.1038/s42003-021-02968-4)
Supplement: Supplementary file 8 — Reporting Summary [file 42003_2021_2968_MOESM8_ESM.pdf]

nature portfolio

Corresponding author(s): Lucas Sire

Last updated by author(s): Nov 30, 2021

Reporting Summary

Nature Portfolio wishes to improve the reproducibility of the work that we publish. This form provides structure for consistency and transparency in reporting. For further information on Nature Portfolio policies, see our Editorial Policies and the Editorial Policy Checklist.

Statistics

For all statistical analyses, confirm that the following items are present in the figure legend, table legend, main text, or Methods section.

n/a	Confirmed	
☐	☒	The exact sample size (*n*) for each experimental group/condition, given as a discrete number and unit of measurement
☐	☒	A statement on whether measurements were taken from distinct samples or whether the same sample was measured repeatedly
☐	☒	The statistical test(s) used AND whether they are one- or two-sided<br>*Only common tests should be described solely by name; describe more complex techniques in the Methods section.*
☐	☒	A description of all covariates tested
☐	☒	A description of any assumptions or corrections, such as tests of normality and adjustment for multiple comparisons
☐	☒	A full description of the statistical parameters including central tendency (e.g. means) or other basic estimates (e.g. regression coefficient) AND variation (e.g. standard deviation) or associated estimates of uncertainty (e.g. confidence intervals)
☐	☒	For null hypothesis testing, the test statistic (e.g. *F*, *t*, *r*) with confidence intervals, effect sizes, degrees of freedom and *P* value noted<br>*Give P values as exact values whenever suitable.*
☒	☐	For Bayesian analysis, information on the choice of priors and Markov chain Monte Carlo settings
☐	☒	For hierarchical and complex designs, identification of the appropriate level for tests and full reporting of outcomes
☒	☐	Estimates of effect sizes (e.g. Cohen's *d*, Pearson's *r*), indicating how they were calculated

Our web collection on statistics for biologists contains articles on many of the points above.

Software and code

Policy information about availability of computer code

Data collection	No software was used to collect data.
Data analysis	All dataset used for analyses and supplementary data for the manuscript are publicly available on Figshare and at the GitHub repository given in the "Data availability" section of the manuscript. DAMe bioinformatic pipeline and R analytic scripts are publicly available on Zenodo and at the GitHub repository referenced in the "Code availability" section of the manuscript. All package/software versions used have been referenced in the "Methods" section. Raw sequencing data from Illumina MiSeq are uploaded to NCBI and are publicly available.

For manuscripts utilizing custom algorithms or software that are central to the research but not yet described in published literature, software must be made available to editors and reviewers. We strongly encourage code deposition in a community repository (e.g. GitHub). See the Nature Portfolio guidelines for submitting code & software for further information.

Data

Policy information about availability of data

All manuscripts must include a data availability statement. This statement should provide the following information, where applicable:

- Accession codes, unique identifiers, or web links for publicly available datasets
- A description of any restrictions on data availability
- For clinical datasets or third party data, please ensure that the statement adheres to our policy

All scripts and datasets used for analyses are publicly available at the following GitHub repository: https://github.com/Lucasire/Malaise_FR_2017 or on Zenodo (https://doi.org/10.5281/zenodo.5653307) and Figshare (https://doi.org/10.6084/m9.figshare.16975636.v1). Raw sequencing data will be available on NCBI upon publication with the following accession number: PRJNA702908.

Field-specific reporting

Please select the one below that is the best fit for your research. If you are not sure, read the appropriate sections before making your selection.

☐ Life sciences ☐ Behavioural & social sciences ☒ Ecological, evolutionary & environmental sciences

For a reference copy of the document with all sections, see nature.com/documents/nr-reporting-summary-flat.pdf

Ecological, evolutionary & environmental sciences study design

All studies must disclose on these points even when the disclosure is negative.

Study description	To understand the effects of climate-induced forest diebacks and subsequent salvage logging on insect biodiversity, we sampled insects during 2017 growing season using non-selective passive-sampling traps and performed metabarcoding on the samples to bypass taxonomic impediment and characterize our communities. a total of 56 forest plots were surveyed and categorized following a stand type (healthy, disturbed, salvaged) and a dieback gradient (low, medium, high) to assess both the impacts of the disturbances per se and the severity of diebacks. To analyze our metabarcoding results, we performed Mvabund analyses, Multi-Site Generalized Dissimilarity Modelling of zeta diversity and Joint Species Distribution Modelling.
Research sample	To assess the response of insect communities to forest disturbances, the sample units are the forest plots. Data from different months were pooled per plot. Hence, a sample unit is considered to represent the insect community at the given study site. However, for accumulation curves, species richness extrapolations and temporal turnover analyses, each time-series of all plot are considered as sample units.
Sampling strategy	Passive-sampling using Malaise traps filled with 80% pure ethanol/20%mono-propylene glycol was performed from mid-May to mid-September 2017 as it represents the best emergence period to sample most aerial insects. No statistical methods were used to predetermine sample size.
Data collection	Environmental data collected on-site where recorded by Laurent Larrieu (co-author) and his team of trained technicians (acknowledged in the according section of the manuscript). Data were recovered on-site by at least 2 experimenters and backed on 2 computers until use in statistical analyses.
Timing and spatial scale	Insect sampling was performed monthly from May 15 to September 15 2017. This gave 4 temporal replicates for each of the 56 plots (224 samples) distributed in Eastern and Central French Pyrenees. This periodicity was chosen to facilitate the recovery of all the Malaise trap samples on-site within a week for each sampling session. The full time period correspond to the seasonal emergence period of most aerial insects.<br>Environmental variables for each plot were collected throughout September 2017.
Data exclusions	No data was excluded from the analysis.
Reproducibility	Sampled sites have been listed with their geographical coordinates in a supplementary list for potential re-sampling. Remaining bodies of insects larger than a bee deriving from the homogenization-size step are kept dry in 50-mL Falcon tubes at -25°C as morphological back-up. Powders resulting from insect grinding of each Malaise trap time-point are stored at -25°C.<br>Similarly, all DNA extracts resulting from the subsampling of these powders and processed until sequencing are stored at -25°C.<br>Methods are fully described in the manuscripts (with software versions whenever applicable) and scripts and datasets to reproduce analyses publicly available.
Randomization	Randomization of samples' processing order (both for sites and time-points of each site) was performed during all laboratory steps to avoid experimenter biases.
Blinding	Experimenters at the laboratory were not aware of the disturbance nature of the plots that the samples were deriving from.

Did the study involve field work? ☒ Yes ☐ No

Field work, collection and transport

Field conditions	Study sites corresponded to mountain (steep) plots of silver fir-dominated forests. Overall, summer conditions (few to no rainfall and warm weather from 20-30°C) were observed during sampling period.
Location	Sampling was performed in Aure valley and Sault plateau in the French Pyrenees. A full list of the 56 plots and their geographic coordinates as well as level of forest disturbances is avalaible in supplementary information. Environmental variables (altitude included) can be found in the publicly available file of environmental data in the aforementioned GitHub repository.
Access & import/export	Collecting was carried out outside nature reserves in both public and private land. Permission from private owners and national forest office was obtained to set up the Malaise traps in their land. No import or export permits were required since sampling was carried out within France
Disturbance	Passive insect sampling with one Malaise trap per site only was performed to reduce impact on communities and to minimize habitat destruction compared to active sampling methods.

Reporting for specific materials, systems and methods

We require information from authors about some types of materials, experimental systems and methods used in many studies. Here, indicate whether each material, system or method listed is relevant to your study. If you are not sure if a list item applies to your research, read the appropriate section before selecting a response.

Materials & experimental systems

n/a	Involved in the study
☒	☐ Antibodies
☒	☐ Eukaryotic cell lines
☒	☐ Palaeontology and archaeology
☐	☒ Animals and other organisms
☒	☐ Human research participants
☒	☐ Clinical data
☒	☐ Dual use research of concern

Methods

n/a	Involved in the study
☒	☐ ChIP-seq
☒	☐ Flow cytometry
☒	☐ MRI-based neuroimaging

Animals and other organisms

Policy information about studies involving animals; ARRIVE guidelines recommended for reporting animal research

Laboratory animals	The study did not involve laboratory animals.
Wild animals	Nearly 3000 different MOTUs (species proxy) of wild insects were passively caught and killed in ethanol-filled jars of Malaise traps. Sampling jars were then transported by car from the study site to the laboratory and stored at 6°C until laboratory processing. Insects were then grinded into powder for subsample DNA extraction, with the remaining powder being kept as back-up at -25°C.
Field-collected samples	Field-collected samples (insect bulks from Malaise traps in 80% pure ethanol / 20% mono-propylene glycol solution) were stored at 6°C except for short time period during transport, until laboratory processing.
Ethics oversight	No ethical approval was required for the sampling of insects as study organisms.

Note that full information on the approval of the study protocol must also be provided in the manuscript.
